# Separation and Characterization of Highly Charged Polyelectrolytes Using Free-Solution Capillary Electrophoresis

**DOI:** 10.3390/polym10121331

**Published:** 2018-12-02

**Authors:** Isabelle Desvignes, Joseph Chamieh, Hervé Cottet

**Affiliations:** Institut des Biomolécules Max Mousseron, University of Montpellier, CNRS, ENSCM, 34095 Montpellier, France; isabelle.desvignes@umontpellier.fr (I.D.); joseph.chamieh@umontpellier.fr (J.C.)

**Keywords:** polyelectrolytes, capillary electrophoresis, charge-density distribution, chemical composition, counterion condensation

## Abstract

The characterization of statistical copolymers of various charge densities remains an important and challenging analytical issue. Indeed, the polyelectrolyte (PE) effective electrophoretic mobility tends to level off above a certain charge density, due to the occurrence of Manning counterion condensation. Surprisingly, we demonstrate in this work that it is possible to get highly resolutive separations of charged PE using free-solution capillary electrophoresis, even above the critical value predicted by the Manning counterion condensation theory. Full separation of nine statistical poly(acrylamide-co-2-acrylamido-2-methylpropanesulfonate) polymers of different charge densities varying between 3% and 100% was obtained by adjusting the ionic strength of the background electrolyte (BGE) in counter electroosmotic mode. Distributions of the chemical charge density could be obtained for the nine PE samples, showing a strong asymmetry of the distribution for the highest-charged PE. This asymmetry can be explained by the different reactivity ratios during the copolymerization. To shed more light on the separation mechanism, effective and apparent selectivities were determined by a systematic study and modeling of the electrophoretic mobility dependence according to the ionic strength. It is demonstrated that the increase in resolution with increasing BGE ionic strength is not only due to a closer matching of the electroosmotic flow magnitude with the PE electrophoretic effective mobility, but also to an increase of the dependence of the PE effective mobility according to the charge density.

## 1. Introduction

The complete analysis of synthetic copolymers requires the use of various experimental techniques, because their heterogeneity along multiple dimensions (molar mass and composition) complicates their characterization. The range of chemical compositions (proportion of each type of monomer in the chain) arises from monomer feed composition drift during the polymerization. While the average composition can be readily determined using, e.g., NMR, no convenient method yields the corresponding sample interpolymer heterogeneity. Such information is especially desirable when some components of the compositional distribution have a large influence on polymer properties. This is particularly likely when one of the monomers is charged, such that the distribution of compositions is equivalent to a distribution of polymer charge densities.

Analyses of polymer distributions invariably require some separation method. Size-exclusion chromatography, which is undoubtedly the most popular polymer separation technique, yields the distribution of polymer hydrodynamic radii or molar masses [1]. Other chromatographic techniques such as interaction chromatography or chromatography at the critical point of adsorption can be useful as well [2]. More recently, attention was focused on capillary electrophoresis (CE) because of its success in the analysis of biopolymers (DNA, proteins, peptides, and polysaccharides), as well as charged synthetic polymers [3,4,5,6,7]. Different modes of CE, including free-solution CE, CE in entangled polymer solutions or gels, and micellar electrokinetic chromatography, were implemented for synthetic polymer analysis, depending on the polymer characteristics (end-charged, evenly charged, or uncharged polymers) and the characteristics of the polymer distribution (distributions of size, functionality, or chemical composition) [7].

Free-solution CE separates polyelectrolytes (PE) according to their linear charge density, independently of the PE molar mass, at least for chain lengths above a few kDa [8]. However, according to counterion condensation theory [9], such separation for vinylic polymers should be possible only if the chemical charge density (molar fraction of repeating units bearing a charge, noted *f*) is lower than ca. 0.37 for vinylic PE, the condition at which the Manning dimensionless charge density parameter *ξ* is below its critical value of unity. Indeed, for *ξ* > 1, counterion condensation occurs and the effective charge density (as well as the effective electrophoretic mobility) should theoretically no longer depend on *f*. This behavior was roughly observed by Hoagland et al. [10] for acrylic acid/acrylamide copolymers, and by Gao et al. [11] and Cottet et al. [12] for random copolymers (PAMAMPS) of 2-acrylamido-2-methylpropanesulfonate (AMPS) and acrylamide (AM). However, all groups experimentally found a weak dependence of the electrophoretic mobility with *f*, even above the 0.37 critical value (*ξ* > 1). While this observation might suggest refinements to the Manning theory, the relationship between electrophoregram peak shape and compositional heterogeneity was pointed out in a few cases. Zhang et al. [13] suggested that broad electropherograms of some commercial samples of sodium poly(styrenesulfonate) could reflect polydispersity in the degree of sulfonation, a consequence of dissolution of polystyrene during sulfonation. Kok et al. [14] used the dependence of the mobility on the degree of substitution (DS) of carboxymethylcellulose to estimate the polydispersity with respect to DS from the electropherograms. Peric et al. [15] suggested that the electrophoregram peak width indicated polydispersity of monomer compositions for acrylic acid/acrylamide copolymers. Staggemeier et al. [16] obtained distributions of *f* from frontal analysis continuous capillary electrophoresis electropherograms of PAMAMPS copolymers. More recently, the correct way of extracting the so-called mass-weighted probability density function, relative to the PE charge density distribution from the time-scale (raw) electropherogram, was addressed [17]. This methodology, relative to the data processing of the electropherograms, takes into account the differences in migration velocities between polymer solutes (which is inherent to any electrophoretic migration), and the mathematical transformations performed on two axes when changing the variable from time to effective mobility [17]. The comparison between different descriptors of the compositional polydispersity was also recently published and applied to the characterization of chitosans [18,19].

In this work, the optimization of the electrophoretic separation of variously charged copolymers according to the ionic strength of the background electrolyte (BGE) was addressed taking PAMAMPS as model compounds. To widen and develop CE applications, it is of major concern to improve the selectivity/resolution of the electrophoretic separation according to the charge density, especially for PE having high charge density. This is a challenging issue since, as discussed earlier, the dependence of the effective mobility on the charge density becomes extremely weak above the Manning critical linear charge density. The ionic strength is one of the major optimizing parameters in CE, which may affect both the polyelectrolyte effective mobility and the electroosmotic mobility and, thus, may have a major impact on the resolution of the separation.

## 2. Materials and Methods

**Chemicals.** Sodium tetraborate decahydrate was obtained from Fluka Chemika (Buchs, Germany). Mesityl oxide was provided by Avocado (La Tour du Pin, France), while 2-acrylamido-2-methyl-1-propane sulfonic acid (AMPS), acrylamide (AM), *N*,*N*,*N*’,*N*’-tetramethylethyldiamine, and potassium persulfate were purchased from Sigma Aldrich (Steinheim, Germany). Deionized water was further purified with a Milli-Q-system from Millipore (Molsheim, France).

**Polymers.** Random copolymers (PAMAMPS) of acrylamide (AM) and 2-acrylamido-2-methylpropanesulfonate (AMPS) were synthesized to high conversion, at room temperature, via radical polymerization initiated by potassium persulfate and *N*,*N*,*N*’,*N*’-tetramethylethylenediamine (TMEDA) according to the procedure described by McCormick [20]. Copolymers were prepared using monomer feeds with AMPS mole fractions of 3%, 5%, 10%, 15%, 20%, 30%, 40%, 55%, 70%, 85%, and 100%. The mean composition of the copolymer *f* measured using proton NMR (3.75 g/L polymer solution in D_2_O) was close to the monomer feed compositions, indicating high conversion. The average molecular masses of the PAMAMPS were evaluated using size-exclusion chromatography coupled with multi-angle laser light scattering (SEC–MALLS), and ranged between 3 × 10^5^ and 10^6^ g/mol [21]. For such high molar masses, the PE electrophoretic mobility is independent of the molar mass (free-draining behavior [8,12,22]). For more details on the synthesis and characterization of the PAMAMPS used in this work, the reader can refer to Reference [21].

**Capillary Electrophoresis Instrumentation.** Capillary electrophoresis (CE) was carried out either with an Agilent Technologies CE system (Waldbronn, Germany) equipped with diode-array detector or a PACE MDQ Beckman Coulter (Fullerton, CA, USA) apparatus. Separation capillaries prepared from bare silica tubing were purchased from Composite Metal Services (Worcester, UK). Capillary dimensions were 33.5 cm (25 cm to the detector) × 50 µm inner diameter (ID) for the Agilent instrument and 30 cm (20 cm to the detector) × 50 µm ID for the Beckman Coulter instrument. New capillaries were conditioned by performing the following washes (20 psi): 1 M NaOH for 15 min, 0.1 M NaOH for 15 min, and the electrolyte for 10 min. In between two runs, the capillary was successively washed by 0.1 M NaOH for 3 min and the electrolyte for 2 min. Sample volumes of approximately 4 nL were introduced hydrodynamically (0.3 psi, 3 s). Sample polymers were prepared, 24 h before analysis, by dissolving the PAMAMPS at 2 g/L in water. If necessary, 0.05% (*v*/*v*) mesityl oxide was added to the sample as a neutral marker. All separations were performed in the positive polarity mode. The temperature of the capillary cassette was maintained constant at 25 °C. Data were collected at 200 nm. For each run, electroosmotic mobility was calculated from the migration time of a neutral marker (mesityl oxide).

Electropherograms were plotted in effective mobility scale by changing the *x*-axis using the following equation:

(1) $$\mu_{ep}^{}\;=\;\left(\frac{1}{t}-\frac{1}{t_{eo}^{}}\right)\frac{lL}{V}$$

where *µ_ep_* is the effective mobility, *l* is the effective capillary length to the detection point, *L* is the total capillary length, *V* is the applied voltage, *t_eo_* is the detection time of the neutral marker, and *t* is the migration time. The *y*-axis used for the effective mobility-scale electropherogram was calculated using the following equation [17]:

(2) $$j\left(\mu_{ep}^{}\right)\;=\;A(t)\times t$$

where *A*(*t*) is the recorded absorbance.

The weight- or mass-average effective mobility was obtained for each PE sample by integration of the mobility-scale distribution *j*(*µ_ep_*) according to

(3) $$\;\bar{\mu_{ep}\;}=\;\frac{\int j\left(\mu_{ep}^{}\right)\mu_{ep}^{}d\mu_{ep}^{}}{\int j\left(\mu_{ep}^{}\right)d\mu_{ep}^{}}\;$$

Corrections of the electrophoretic mobility from Joule heating were performed using a previously described procedure [23] according to the following equation:

(4) $$\bar{\mu_{ep,corr}}\;=\;\frac{\bar{\mu_{ep}}}{1\;+\;\alpha \frac{P_{w}}{L}}\;,$$

where *P_W_* is the dissipated power, and the factor ⍺ corresponds to the slope of the relative variation of the conductivity (κ*_(P)_*/κ*_(P=_*_0*)*_) as a function of the dissipated power per unit of length (*P_W_*/*L*) determined at 25 °C on the CE equipment (*⍺* = 0.07 on Agilent and *⍺* = 0.04 on Beckman Coulter). In all experiments performed to determine the effective PE mobility, the applied voltage was chosen so as to keep *P_W_/L* values lower than 0.7 W·m^−1^ (i.e., corresponding to an increase of temperature of less than 2 °C).

## 3. Results and Discussion

**Figure 1:**
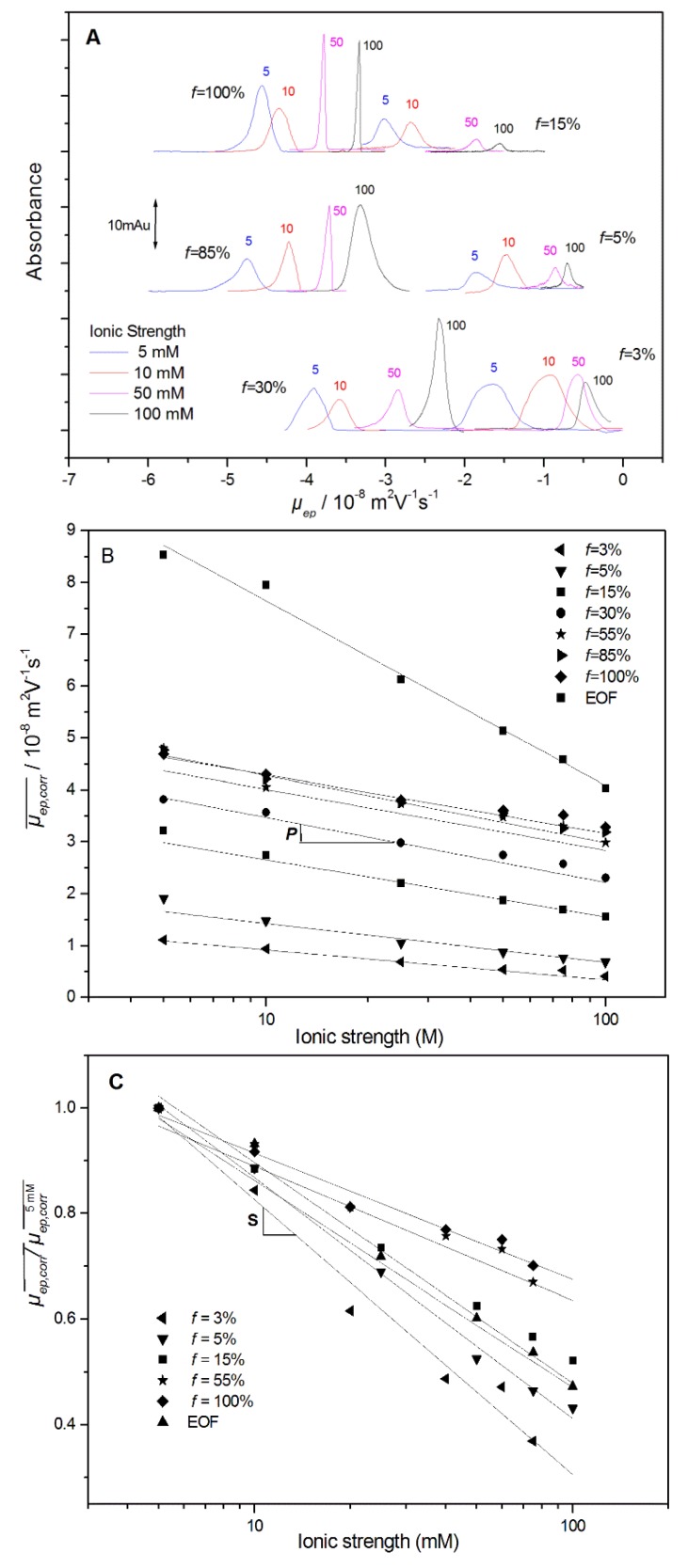
Effective mobility-scale electropherograms obtained for random copolymers (PAMAMPS) of 2-acrylamido-2-methylpropanesulfonate (AMPS) and acrylamide (AM) of different charge densities and at different ionic strengths (**A**), and the corresponding ionic strength dependences of the effective mobility $\bar{\mu_{ep,corr}}$ (**B**) or $\frac{\bar{\mu_{ep,corr}}}{\bar{\mu_{ep,corr}^{5\;mM}}}$ (**C**). Electrophoretic conditions: fused silica capillary, 33.5 cm (25 cm to the detector) × 50 µm inner diameter. Electrolyte: sodium borate buffer at the ionic strength as indicated on the graph, pH 9.2. Applied voltage: +5 kV at 50 mM ionic strength and above, +10 kV otherwise. Sample: 2 g/L of each copolymer in water. Hydrodynamic injection: 17 mbar, 3 s. Ultraviolet detection at 200 nm. Temperature: 25 °C. Peak identification: AMPS mole content (*f*) in the copolymer as indicated. EOF: electroosmotic flow.

**Figure 2:**
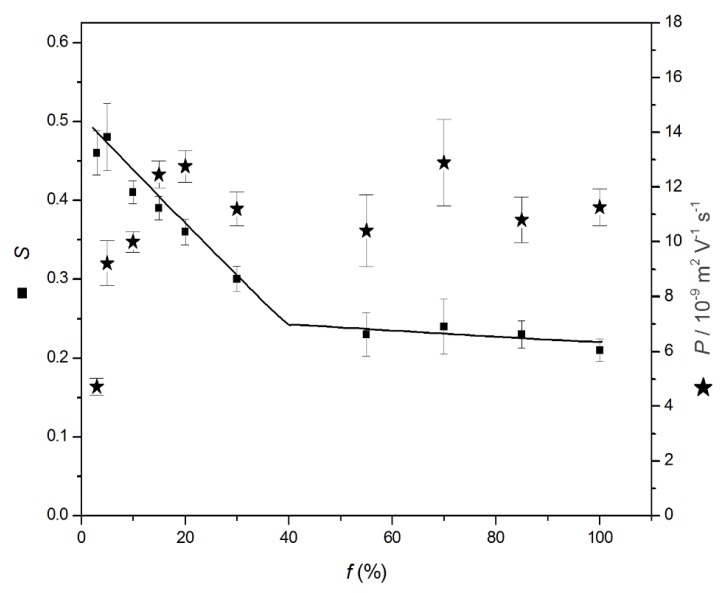
Variations of the *P* and *S* values according to the PAMAMPS chemical charge density *f*. Experimental conditions as in Figure 1. Error bars are ± one standard deviation on the *S* or *P* slopes from graphs in Figure 1B,C. Lines are only guides for the eyes.

**Ionic Strength Dependence of the PE Electrophoretic Mobility.** Before looking at the separation of mixtures of PE of different charge densities, the electrophoretic behavior of PE according to the ionic strength was firstly investigated by injecting each PE individually. Figure 1A displays the mobility-scale electropherograms obtained for different PAMAMPS with *f* varying between 3% and 100%, at different ionic strengths from 5 mM up to 100 mM, in sodium borate BGE at pH 9.2. The average effective electrophoretic mobilities were calculated through the integration of the mobility scale electropherograms [17], and corrected from Joule heating as described in Section 2. The dependence of the PE effective electrophoretic mobility with the ionic strength *I* (in M) is displayed in Figure 1B, including the fitting curves according to the following equation:

(5) $$\bar{\mu_{ep,corr}}=-P\log(I)+\mu_{ep,extr}^{1M},$$

where *P* is the effective mobility decrease per ionic strength decade, and $\mu_{ep,extr}^{1M}$ is the extrapolated PE effective mobility at 1 M ionic strength. Logarithmic dependence of the effective mobility with the ionic strength was reported either for random-coil or long-rod PE conformations [24]. Such a representation presents the advantage of quantifying the ionic strength dependence on the 5–100 mM range and permitting the interpolation of the effective mobility values at any ionic strength [25]. In practice, the *P* value corresponds to the PE effective mobility decrease per ionic strength decade. As displayed in Figure 2, *P* increases rapidly at low charge density from *P* ~4.7 TU at *f* = 3%, up to *P* ~10–11 TU for *f* ≥ 10%, and becomes independent of *f*, where TU stands for Tiselius unit (1 TU = 10^−9^ m^2^·V^−1^·s^−1^). All numerical *P* values are given in the Supplementary Materials (see Table S1). Interestingly, the *P* value (~30–40 TU) observed for the electroosmotic flow (EOF) is much higher than that for the PE, which means that the apparent mobility of the PE decreases with the ionic strength, as discussed in more detail in the next section. The ionic strength dependence can be also plotted in terms of a relative decrease compared to a value of reference, arbitrarily taken at 5 mM ionic strength $\bar{\mu_{ep,corr}^{5\;mM}}$, according to the following equation [25]:

(6) $$\frac{\bar{\mu_{ep,corr}}}{\bar{\mu_{ep,corr}^{5\;mM}}}=-S\log(I)+\frac{\mu_{ep,extr}^{1M}}{\bar{\mu_{ep,corr}^{5\;mM}}}.$$

The *S* parameter derived from Equation (6) and from Figure 1C is adimensional and is represented in Figure 2 (numerical values in Table S1, Supplementary Materials). A value of *S* = 0.46, as obtained in this work for the PAMAMPS 3%, means that its effective mobility decreases by 46% of its initial value at 5 mM ionic strength per ionic strength decade. The *S* parameter decreases with *f* until *f* reaches the Manning condensation threshold (*f* = 37%) above which *S* remains almost constant at ~0.23. These figures of merit are very useful for estimating the impact of the ionic strength on the effective mobility and for the modeling of the effective and apparent selectivities according to *f* and *I,* as discussed in the next section. The *S* values are also very informative about the characteristics of the solute (charge, nature, or size) [25,26,27]. As a matter of comparison, proteins have typical *S* values between 0.3 and 0.4 [27], which are very similar to the values obtained for moderately charged PAMAMPS with 30% ≥ *f* ≥ 10%. For small monocharged ions, such as sodium, much lower *S* values of ~0.1 were reported [28], while higher *S* values of ~0.44–0.53 were obtained for electroosmotic flow (EOF) [25].

**Figure 3:**
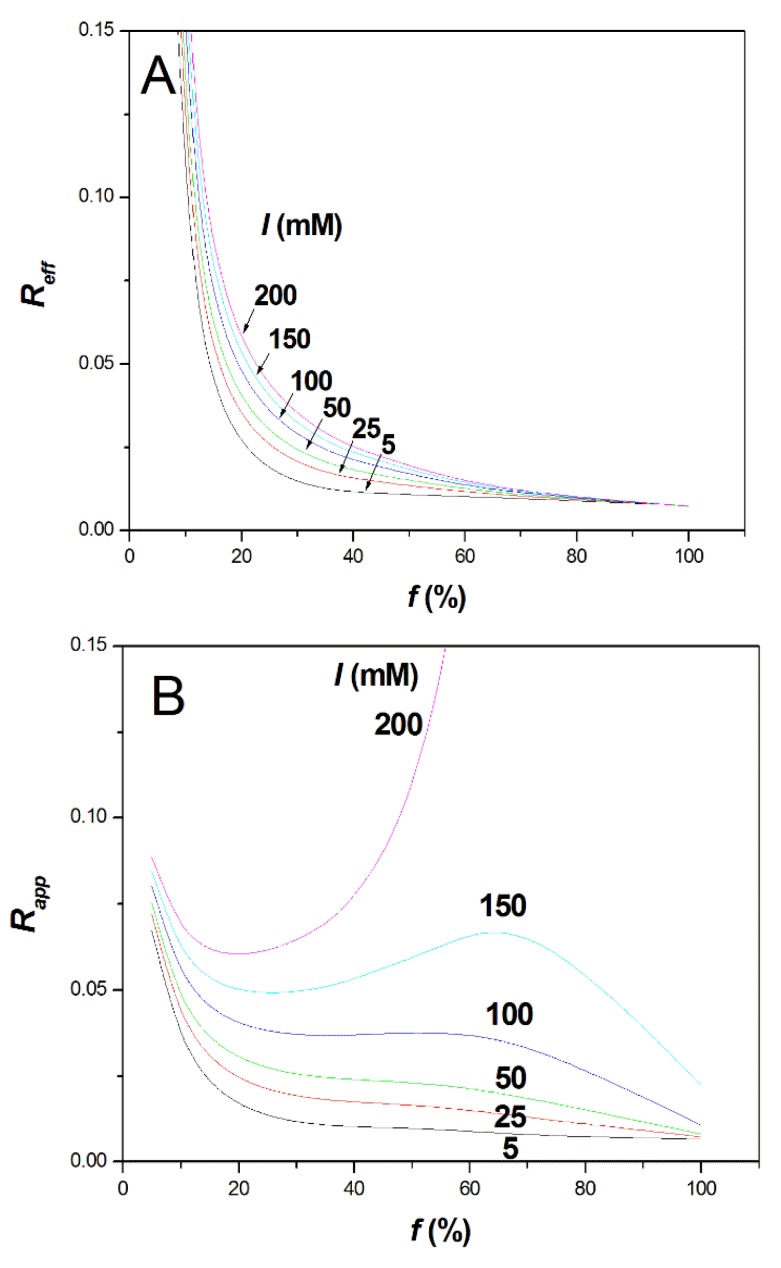
Variation of the effective *R_eff_* (**A**) and apparent *R_app_* (**B**) selectivities for the separation of PAMAMPS copolymers according to the charge density *f* at different ionic strengths from 5 mM to 200 mM. *R_eff_* and *R_app_* were calculated using Equations (7) and (8) based on the numerical values given in Table S1 (Supplementary Materials) derived from experimental data presented in Figure 1. The following empirical equations obtained by curve-fitting of the experimental data were used in Equations (7) and (8): *S_i_* = −0.5883*f*^3^ + 1.2674*f*^2^ − 0.9736*f* + 0.5041; $\mu_{ep,corr,i}^{5\;mM}$ (in Tiselius units (TU)) = −(10.025 × ln*f* + 48.976); $\mu_{ep,corr,i}^{1M}$ (in TU) = −(9.9693*f*^3^ − 42.714*f*^2^ + 59.236*f* − 2.299); *S_eo_* = 0.4168; $\mu_{eo,corr}^{5\;mM}$ = 85.3 TU; $\mu_{eo,corr}^{1M}$ = 5.24 TU.

**Predicting the Change in Selectivity between Two PEs of Different Charge Density According to the Ionic Strength.** The effective selectivity *R_eff_* and the apparent selectivity *R_app_* between two PAMAMPS with closely related charge densities *f*_1_ and *f*_2_, are respectively defined as [29]:

(7) $$R_{eff}=\left|\frac{2\;\Delta \bar{{µ}_{ep,corr}}}{\bar{{µ}_{ep,corr,1}}+\bar{{µ}_{ep,corr,2}}}\right|≃\left|\frac{\Delta \bar{{µ}_{ep,corr}}}{\bar{{µ}_{ep,corr,1}}}\right|,$$

and

(8) $$R_{app}=\left|\frac{2\;\Delta \bar{{µ}_{ep,corr}}}{\bar{{µ}_{app,corr,1}}+\bar{{µ}_{app,corr,2}}}\right|≃\left|\frac{\Delta \bar{{µ}_{ep,corr}}}{\bar{{µ}_{ep,corr,1}}+\;{µ}_{eo,corr}}\right|,$$

where $\bar{{µ}_{ep,corr,i}}$ and $\bar{{µ}_{app,corr,i}}$ are the Joule-heating-corrected effective and apparent mobilities of polyelectrolyte *i*, ${µ}_{eo,corr}$ is the Joule-heating-corrected electroosmotic mobility, and $\Delta \bar{{µ}_{ep,corr}}$ is the difference in effective mobility between the two PEs of different charge densities *f*_1_ and *f*_2_. In Equation (8), mobilities are algebraic numbers (i.e., positive for the EOF, and negative for the anionic PAMAMPS). Combining Equations (6)–(8), *R_app_* and *R_eff_* can be expressed as functions of the ionic strength *I*, the *S* parameters, and the effective mobilities at 5 mM and 1 M ionic strength of each polyelectrolyte, as:

(9) $$R_{eff}(I)=\frac{\left(S_{1}\bar{\mu_{{}_{ep,corr,1}}^{5mM}}-S_{2}\bar{\mu_{{}_{ep,corr,2}}^{5mM}}\right)\log I+\mu_{{}_{ep,extr,2}}^{1M}-\mu_{{}_{ep,extr,1}}^{1M}}{{µ}_{{}_{ep,extr,1}}^{1M}-S_{1}\bar{\mu_{{}_{ep,corr,1}}^{5mM}}\log I},$$

and

(10) $$R_{app}(I)=\frac{\left(S_{1}\bar{\mu_{{}_{ep,corr,1}}^{5mM}}-S_{2}\bar{\mu_{{}_{ep,corr,2}}^{5mM}}\right)\log I+\mu_{{}_{ep,extr,2}}^{1M}-\mu_{{}_{ep,extr,1}}^{1M}}{{µ}_{{}_{eo,corr}}^{1M}+{µ}_{{}_{ep,extr,1}}^{1M}-\left(S_{eo}\bar{\mu_{{}_{eo,corr}}^{5mM}}+S_{1}\bar{\mu_{{}_{ep,corr,1}}^{5mM}}\right)\log I},$$

where *S_i_* is the *S* parameter for polyelectrolyte *i*, and the superscript on the mobility refers to the ionic strength. Taking *f* = *f*_2_ − *f*_1_ = 2% and using the numerical values given in Table S1 (Supplementary Materials) and the caption of Figure 3, the effective selectivity and apparent selectivity are plotted in Figure 3A,B as a function of *f* for different ionic strengths. The numerical values obtained for the selectivities are not informative in absolute value since they obviously depend on the constant value arbitrarily taken for *f* to plot Figure 3. However, the trends in the dependence of selectivities with *f* and with *I* are instructive.

At low ionic strength (*I* = 5 mM), the effective selectivity dramatically drops with increasing *f* and becomes almost constant above the Manning condensation threshold (i.e., for *f* typically higher than 36%), as shown in Figure 3A. Between *f* = 10% and *f* > 37%, the effective selectivity drops by a factor of ~10. Interestingly, increasing the ionic strength to about 100–200 mM is beneficial to the effective selectivity, since it tends to extend the selectivity drop over a broader range of *f* before becoming constant above typically *f* ≈ 60%. Therefore, intrinsically, the increase of ionic strength tends to increase the effective selectivity of highly charged PE, even above the Manning condensation threshold. However, for *f* ≥ 60%, the effective selectivity remains very small, whatever the ionic strength. It is not clear if the weak dependency of the polyelectrolyte electrophoretic mobility with the charge density is due to a breakdown of the Manning theory, or if it is related to specificities of the polyelectrolyte chain. The Manning theory assumes that the charges are evenly distributed along the chain. Therefore, intra-chain heterogeneity with a drift of composition along the chain, possibly combined with “end-effects”, could be at the origin of this weak dependency of the electrophoretic mobility according to *f*. The “end-effects” mean that the ends of the chain are hydrodynamically more exposed to the solvent than the center of the chain. This effect was, for instance, evocated to explain non-zero mobility of an overall neutral polyampholyte chain [30]. The hypothesis on intra-chain heterogeneity is, however, very difficult to assess since, unfortunately, we cannot perfectly synthesize evenly charge-distributed copolymers.

As for the apparent selectivity, which takes into account the EOF and the migration in counter-electroosmotic mode, it is remarkable to notice that, by counter balancing the PE electrophoretic migration with the EOF, it is possible to considerably increase the apparent selectivity, even for the low effective selectivities observed at high *f* values (i.e., for *f* typically higher than 60–70%). At 0.15 M ionic strength, the selectivity obtained at *f* ≈ 65% becomes similar to the apparent selectivity observed at low *f* ≈ 5%. Of course, this huge increase of selectivity has to be paid by an important increase of the migration time and, thus, of the analysis time. However, the possibility of overcoming the intrinsic low selectivity due to the Manning counterion condensation through the counter EOF mode constitutes a unique opportunity to improve the characterization of highly charged PE using free-solution CE. The counter EOF mode is known to especially improve the resolution of the late-migrating solutes. It is, therefore, well suited to improve the apparent selectivity of the most densely charged polyelectrolytes, i.e., those for which the effective selectivity is lower.

**Figure 4:**
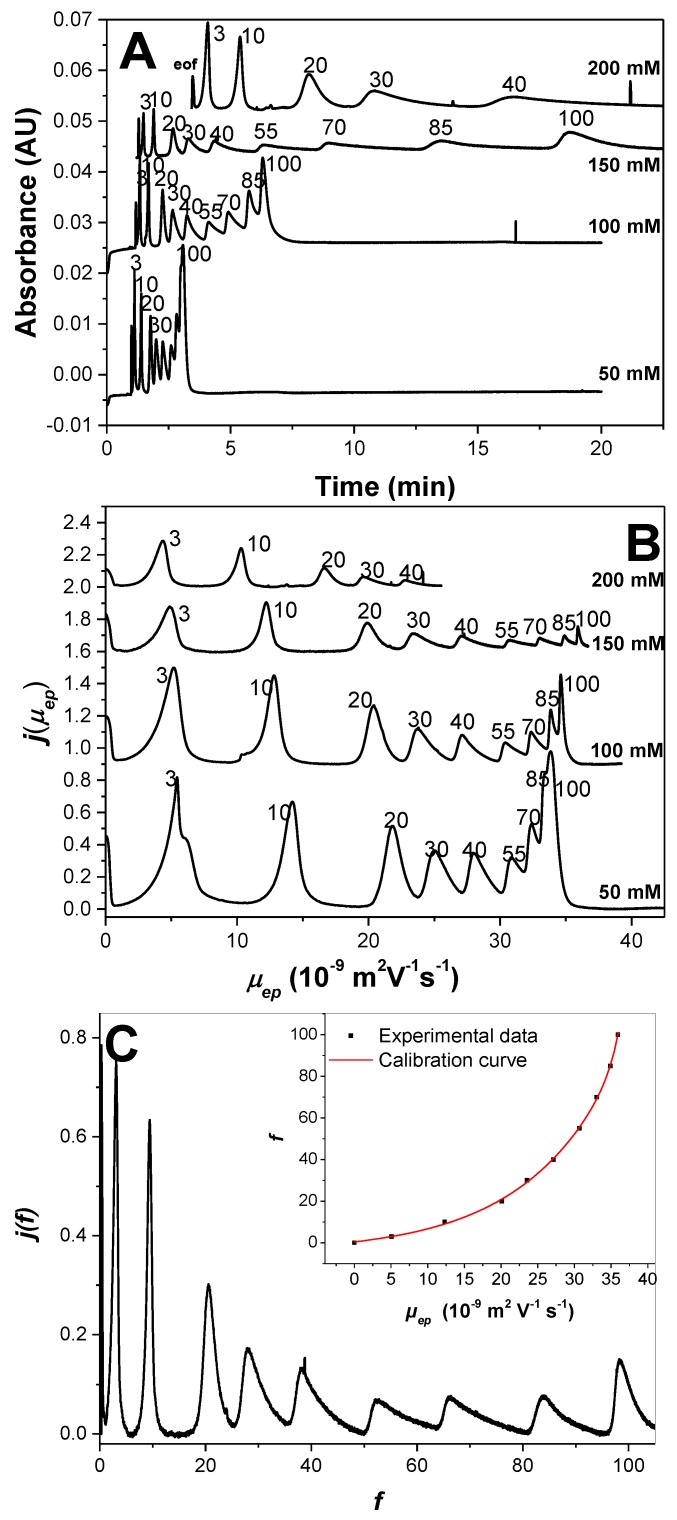
Influence of the background electrolyte (BGE) ionic strength on the separation of nine PAMAMPS copolymers according to their charge density. Raw time-scale electropherograms (**A**) and effective mobility-scale distributions (**B**) and charge-density distribution at 150 mM ionic strength (**C**). Electrophoretic conditions: fused silica capillary, 30 cm (20 cm to the detector) × 50 µm inner diameter. Electrolyte: sodium borate buffer with borate concentration corresponding to two times the ionic strength indicated on the graphs, pH 9.2. Applied voltage: +20 kV, except at 200 mM ionic strength (+10 kV). Sample: 2 g/L of each copolymer in water. Hydrodynamic injection: 0.3 psi, 3 s. Ultraviolet detection at 200 nm. Temperature: 25 °C. Peak identification: AMPS mole content in the copolymer as indicated. EOF: electroosmotic flow. Insert of Figure 4C: correlation (calibration curve) between *f* and the PAMAMPS effective mobility according to the following empirical equation: $f=-6.7+7.02\times \exp(0.07\times \mu_{ep})+1.81\times 10^{-6}\times \exp(0.46\times \mu_{ep})$, where *µ_ep_* is in TU and *f* is in %.

**Application to the Optimal Separation of Nine Variously Charged PAMAMPS and to the Achievement of the Entire Charge Distribution.** To demonstrate the possibility of separating PEs above the Manning condensation threshold, the separation of nine PAMAMPS of different charge densities (*f* = 3%, 10%, 20%, 30%, 40%, 55%, 70%, 85%, and 100%) was performed in sodium borate buffers (pH 9.2) of different ionic strengths ranging between 50 mM and 200 mM (100 and 400 mM borate concentrations, respectively). Separations are displayed in Figure 4 in the time scale (Figure 4A) and effective mobility scale (Figure 4B). Figure 4A clearly demonstrates that the resolution of the CE separation according to *f* increases with increasing ionic strength. At 200 mM ionic strength, the EOF becomes too slow to allow the detection of the most highly charged PE within a reasonable time. After 25 min of analysis, only PEs with *f* ≤ 40% were detected. Ionic strength at 150 mM appeared to be a good compromise between resolution and analysis time, with all nine PEs almost fully resolved, even those with *f* > 0.37, within about 25 min. It is remarkable that the PE separation can be obtained, even above the Manning condensation threshold, and up to *f* = 100%. Such high resolutions were not observed, nor expected, for high-charge-density PE [8,10,12]. These experimental results demonstrate that even low effective selectivity can lead to high apparent selectivity in counter-electroosmotic mode with sufficient resolution to get the charge-density distribution of the nine individual PEs. It is well known that the apparent selectivity in the presence of EOF (separation of anions on a fused silica capillary in counter-electroosmotic mode) is higher that the effective selectivity in the absence of EOF (separation on a neutrally coated capillary), as long as *µ_eo_* is lower than $\left|2\times {µ}_{ep}\right|$ [31]. In the investigated experimental conditions, these conditions were verified at all investigated ionic strengths for the highest charged PE (*f* > 37%), demonstrating that the counter-electroosmotic mode was beneficial to the separation of these PEs. On the whole, separation is enhanced at higher ionic strength because of (i) a closer matching of the EOF magnitude with the electrophoretic effective mobility of the 100% PAMPS (infinite resolution—with infinite migration time—is theoretically accessible for exactly matching EOF and effective mobilities); and (ii) an increase in the effective selectivity with increasing ionic strength as observed in Figure 3A. At 150 mM ionic strength, the dependence of the effective mobility with *f* above *f* = 0.7 (i.e., well above the Manning limit) is much more evident than at 50 mM ionic strength, as seen in Figure 4B.

Taking the 150 mM ionic strength as the optimal condition of separation of the PAMAMPS mixture, the entire charge-density distribution was plotted in Figure 4C using the data processing described in detail in Reference [17]. In brief, the raw (time-scale) electropherogram was first converted into a mass-weighted distribution of migration times *h*(*t*), by dividing the recorded absorbance *S*(*t*) by the migration time (*h*(*t*) = *S*(*t*)/*t*). Then, this distribution was converted into a mass-weighted effective mobility distribution (*j*(*µ_ep_*) as a function of *µ_ep_*) by changing the *x*-axis from time to effective mobility, and by multiplying the time-corrected absorbance by *t^2^* (*j*(*µ_ep_*) = *h*(*t*) × *t*^2^). Finally, the mass-weighted charge density distribution (*j*(*f*) as a function of *f*) was obtained by changing the *x*-axis from *µ_ep_* into *f* using a correlation between the average effective mobility and the average *f* value obtained by ^1^H NMR. Note that the *f* values were equal to the feed ratio since the polymerization was conducted to complete conversion. The calibration curve is presented in the inset of Figure 4C. The *y*-axis of the desired distribution *j*(*f*) is obtained by dividing *j*(*µ_ep_*) by the first derivative of *f* as a function of *µ_ep_* according to:

(11) $$j(f)=\frac{j(\mu_{ep})\;}{\frac{\partial f}{\partial {µ}_{ep}}}=\frac{h(t)\times t^{2}\;}{\frac{\partial f}{\partial {µ}_{ep}}}=\frac{S(t)\times t\;}{\frac{\partial f}{\partial {µ}_{ep}}}=\frac{S(t)\times t\;}{\left(8.32\times 10^{-7}\times \exp\left(0.46\times {µ}_{ep}\right)+0.49\times \exp\left(0.07{µ}_{ep}\right)\right)},$$

with *µ_ep_* in TU in the numerical expression. The normalized charge-density distributions obtained for the different PAMAMPS samples look much more symmetrical for the low-charge-density PAMAMPS (*f* between 3% and 20%), while a high dissymmetry toward the highest charge density was observed for the high-charge-density PAMAMPS (*f* ≥ 30%). This dissymmetry is directly correlated to the inter-chain charge-density distribution which is controlled by the differences in reactivity factor during the copolymerization. The composition drift in the copolymerization of AM and AMPS is due to reactivity ratios $r_{1}=\frac{k_{11}}{k_{12}}$ = 1.0 and $r_{2}=\frac{k_{22}}{k_{21}}$ = 0.4 [20,32], where the subscript 1 refers to the AM monomer and subscript 2 refers to the AMPS monomer, and the *k* values are the kinetic constants of polymer propagation [33]. These reactivity ratios favor the formation of AMPS-rich copolymer chains with AMPS sequences at high conversion, explaining a tailing of the charge density toward high *f* values. From the analytical point of view, the remarkable distributions obtained in Figure 4C can be used to extract the information about the polydispersity of all PAMAMPS samples. The dispersion of the distributions are presented in Table 1 in terms of standard deviations *σ_f_* or in terms of dispersity indexes (*PDI*), using two different ratios based on various moments of the distribution in charge density *j*(*f*) according to the following equations:

(12) $$PDI\left(j(f),1,0\right)=\frac{\bar{f_{}^{1}}\times \bar{f_{}^{-1}}}{{\left[\bar{f_{}^{0}}\right]}^{2}}=\frac{\left(\int j(f)fdf\right)\times \left(\int \frac{j(f)}{f}df\right)}{{\left(\int j(f)df\right)}^{2}},$$

(13) $$PDI\left(j(f),2,1\right)=\frac{\bar{f_{}^{2}}\times \bar{f_{}^{0}}}{{\left[\bar{f_{}^{1}}\right]}^{2}}=\frac{\left(\int j(f)f^{2}df\right)\times \left(\int j(f)df\right)}{{\left(\int j(f)fdf\right)}^{2}},$$

where $\bar{f_{}^{i}}$ is the *i*st order moment defined as $\int j(f)f^{i}df$. As can be seen from Table 1, the standard deviation of the mass distribution in *f* varies between 0.6 at low charge density (3%) and 3–3.7 at high charge densities (70% or 85%). The corresponding *PDI* varied between 1.001 and 1.049, showing a relatively low polydispersity in charge for the studied polymers, albeit with higher values for lower-charge-density polymers. As can be seen from Table 1, the same tendency is observed for the two *PDI* definitions given by Equations (12) and (13), and for the σ*_f_*/*f_w_* ratio, showing that all of these parameters can be used to quantitatively estimate the polymer dispersity in chemical composition (or charge density).

## 4. Conclusions

The characterization of highly charged statistical copolymers is challenging because of the Manning counterion condensation that greatly decreases the effective selectivity of the separation according to the charge density. The displacement of the Manning counterion threshold toward higher charge density is difficult to obtain experimentally. However, we demonstrate in this work that operating-free solution CE in counter-electroosmotic mode and adjusting the electroosmotic mobility close to the mobility of the highest charged PE by playing on the BGE ionic strength is a very easy and convenient way of optimizing the separation. Surprisingly, highly resolutive separations allowing the full characterization of all PAMAMPS samples, including those with *f* higher than 37%, could be obtained using a BGE at 150 mM ionic strength, within a reasonable analysis time (~25 min). Combined with the recent developments on the data processing of electropherograms for polymer analysis in CE [17,18], this work confirms that CE is a powerful and mature analytical technique for the charge-based characterization of PE, whatever the PE charge density, even for *f* values higher than the Manning condensation threshold. If the counter electroosmotic mode can be used to considerably magnify, in an apparent way, a small effective selectivity, it could not bring any separation if the effective selectivity was null. Therefore, a key point would be to better understand the origin of the small effective selectivity (small increase in effective mobility with *f*) that was observed in this work, and see if any refining theory of counterion condensation could explain it.

## Figures and Tables

**Table 1:** Figures of merit about the characteristics of the charge-density distributions of nine PAMAMPS samples; *f_w_* is the average chemical charge density which was calculated from the integration of each peak in Figure 4 using the calibration curve given in the caption of Figure 4; *σ_w_* is the standard deviation (square root of the second moment of the *f* distribution) of the charge-density distribution, which gives information about the dispersion of the distribution; *σ_w_/f_w_* allows estimating the relative polydispersity of the polymers. *PDI (j(f), i, i + 1)* are dispersity indices defined by Equations (12) and (13).

Sample	*f_w_ (%)*	*σ_w_ (%)*	*σ_w_/f_w_*	*PDI (j(f), 1, 0)*	*PDI (j(f), 2, 1)*
3	2.9	0.6	0.2031	1.049	1.041
10	9.4	0.7	0.0752	1.006	1.006
20	21.0	1.4	0.0665	1.004	1.004
30	29.6	2.2	0.0757	1.006	1.006
40	40.4	2.8	0.0699	1.005	1.005
55	55.8	3.4	0.0616	1.004	1.004
70	69.8	3.7	0.0531	1.003	1.003
85	85.9	2.8	0.0326	1.001	1.001

## Supplementary Materials

The following are available online at http://www.mdpi.com/2073-4360/10/12/1331/s1, Numerical *P* and *S* values with their confidence intervals for PAMAMPS polyelectrolytes are provided in the Supplementary Materials (see Table S1).
