# Supplementary material for: Separation and Characterization of Highly Charged Polyelectrolytes Using Free-Solution Capillary Electrophoresis
Source: Polymers (Basel). 2018 Dec 2;10(12):1331. doi: 10.3390/polym10121331 (PMC6401714; doi:10.3390/polym10121331)
Supplement: Supplementary file 1 [file polymers-10-01331-s001.pdf]

Supplementary Materials: Separation and Characterization of Highly Charged Polyelectrolytes Using Free-Solution Capillary Electrophoresis

Isabelle Desvignes, Joseph Chamieh and Hervé Cottet *

Table SI-1. Numerical *P* and *S* values for PAMAMPS polyelectrolytes.

f (%)	*P* (in TU)	*S*
3	4.72 ± 0.31	0.46 ± 0.01
5	9.21 ± 0.82	0.48 ± 0.05
10	10.00 ± 0.38	0.41 ± 0.02
15	12.46 ± 0.49	0.39 ± 0.02
20	12.76 ± 0.59	0.36 ± 0.02
30	11.20 ± 0.62	0.30 ± 0.02
55	10.4 ± 1.3	0.23 ± 0.03
70	12.9 ± 1.6	0.24 ± 0.04
85	10.80 ± 0.83	0.23 ± 0.02
100	11.26 ± 0.67	0.21 ± 0.02
EOF	30.7 ± 1.7	0.42 ± 0.02
